# Amorphous Silica Interlayer Unlocks Direct Epitaxial Growth of CsPbBr_3_ on Silicon via Slip-and-Stick Mechanism

**DOI:** 10.1021/acs.jpclett.4c03705

**Published:** 2025-02-26

**Authors:** Christian Tantardini, Simone Argiolas, Paola De Padova, Boris I. Yakobson, Aldo Di Carlo, Alessandro Mattoni

**Affiliations:** † CNR - Istituto Officina dei Materiali (IOM) Cagliari, Cittadella Universitaria, Monserrato, Cagliari 09042, Italy; ‡ Department of Materials Science and NanoEngineering, Rice University, Houston, Texas 77005, United States of America; ¶ Dipartimento di Fisica, Università degli Studi di Cagliari, Cittadella Universitaria, Monserrato, Cagliari 09042, Italy; § National Research Council, Institute of Structure of Matter (CNR - ISM), Via Fosso del Cavaliere, 100, 00133 Roma, Italy

## Abstract

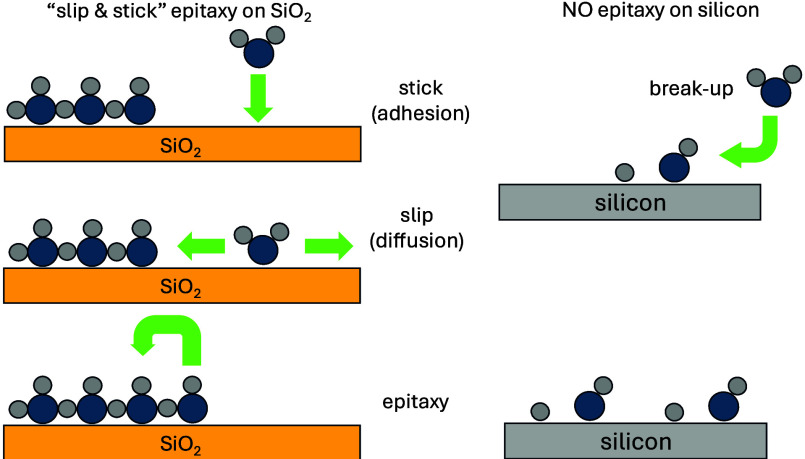

In this study, we investigate the “Slip and Stick” mechanism governing the epitaxial growth of CsPbBr_3_ on amorphous silica surfaces and its implications for silicon/perovskite tandem solar cell applications. The unique, low-energy diffusion behavior of cesium lead bromide on amorphous silica enables molecular species to traverse the surface efficiently without bond-breaking, thereby preserving structural integrity. Consequently, the chemically inert nature of amorphous silica facilitates the formation of crystalline CsPbBr_3_ thin films on silicon substrates, which is essential for tandem solar cell architectures. In contrast, the reactive silicon (111) surface, that induces fragment decomposition and Br doping of Si, poses challenges to device stability due to potential disruptions in structural and electronic continuity. Our findings elucidate the observed difficulties in epitaxially growing metal halide perovskites directly on silicon surfaces and underscore the role of amorphous silica as an ideal passivation layer, promoting the precise layer-by-layer assembly necessary for high-efficiency tandem solar cells.

Lead (Pb) is crucial for endowing perovskite materials with exceptional optoelectronic properties, including direct and tunable band gaps, strong spin–orbit coupling, high absorption coefficients, and efficient light emission.^1,2^ Additionally, lead-based perovskites exhibit remarkable defect tolerance and long charge carrier diffusion lengths, resulting in prolonged carrier lifetimes. These characteristics make lead halide perovskites prime candidates for high-performance optoelectronic devices such as solar cells and light-emitting diodes.^3−8^ Among these materials, the hybrid organic–inorganic methylammonium lead iodide (MAPbI_3_) stands out in photovoltaics for its high solar energy conversion efficiency.^9−13^ However, there is growing interest in all-inorganic perovskites due to their superior stability under high humidity, elevated temperatures, intense ultraviolet (UV) radiation, and mechanical stress,^14−23^ which helps prevent the moisture-induced decomposition and reduced efficiency often seen in hybrid perovskites like MAPbI_3_.^15^

In particular, all-inorganic perovskites such as cesium lead bromide (CsPbBr_3_) avoid volatile organic components, resulting in enhanced moisture resistance and preserved structural integrity at high temperatures.^14,16,17^ They also demonstrate resilience to UV-induced degradation,^18,19^ mechanical robustness for flexible electronics and wearables,^20,21^ and sustained efficiency over extended periods, which is vital for large-scale solar and optoelectronic applications.^22,23^

These stability advantages make all-inorganic perovskites promising candidates for next-generation optoelectronic applications, addressing the limitations of hybrid perovskites and enabling more durable and efficient devices.^5,14^

For example, CsPbBr_3_ possesses a direct band gap of 2.3 eV and have demonstrated ultrahigh electron mobility of 1000 cm^2^V s^–1^ and an electron lifetime of 2.5 μs making it promising for photovoltaic and especially for tandem solar cells. Indeed, the combination of perovskite and silicon can markedly enhance the efficiency of silicon-only devices, reducing energy losses and optimizing energy conversion.^24−26^

Tandem solar cell architectures require precise control of perovskite growth on silicon substrates, as the interface quality critically affects multijunction efficiency.^27^ Among the various perovskites, CsPbBr_3_ stands out for its excellent optoelectronic properties, thermal stability, and suitable band gap for tandem cells,^28,29^ enabling reduced thermalization losses and the potential to surpass single-junction efficiency limits.^30^

Achieving a high-quality CsPbBr_3_–silicon interface is key to minimizing recombination losses and boosting charge carrier extraction.^31^ Recent progress in deposition methods, such as vapor-assisted solution processing and thermal evaporation, demonstrates successful CsPbBr_3_ integration on silicon,^32,33^ while the material’s ambient stability addresses a major challenge of hybrid perovskites, making it appealing for commercial applications.^29^ Further investigation into interface engineering and growth mechanisms is essential for optimizing tandem performance, enhancing efficiency, and extending device lifetimes, bringing highly efficient and stable multijunction solar cells closer to realization.^28,31^

Molecular beam epitaxy (MBE) has emerged as an effective method for achieving atomic-level control over perovskite film growth, enabling the production of thin films with precise stoichiometry, thickness, and crystallinity.^34−45^ This technique is especially well-suited for refining the growth conditions of perovskites on silicon and related substrates, such as amorphous silica (SiO_2_) and crystalline alpha-quartz (α-SiO_2_), which are favored for their compatibility with existing semiconductor technologies and advantageous electronic properties.^34−45^ A thorough control of perovskite growth on these silicon-based substrates could -therefore advance the high-performance of tandem solar cells by improving film quality and interface characteristics.^24−26^ Nevertheless, the epitaxial growth of perovskite on silicon presents a significant challenge. It is noteworthy that recent studies have shown that MBE of CsPbBr_3_ on silicon is possible with the use of a thin amorphous silicon dioxide interlayer.^45^ The underlying microscopic cause of these observations remains unclear and is the object of the present Letter.

The growth mechanisms of perovskite films are highly complex, varying significantly with substrate properties.^34−45^ Research has highlighted two principal growth mechanisms for perovskites: the “slip” mechanism, where perovskite layers laterally slide across smooth surfaces for even film coverage, and the “sticking” mechanism, where the material adheres to the substrate, accumulating in areas with surface roughness.^45−48^

Recently, a combined “slip-and-sticking” mechanism has been proposed, integrating both lateral sliding and substrate adhesion to enhance film uniformity and crystallinity.^49−52^ In this combined mechanism, the initial sticking facilitates nucleation and anchoring of perovskite crystals on the substrate, particularly in regions with inherent surface irregularities. Subsequently, the slip component allows the perovskite layers to spread laterally across the substrate, promoting even coverage and reducing defects. This synergistic interplay ensures that the film achieves high uniformity while maintaining strong adhesion to the substrate, which is crucial for the performance and stability of perovskite-based devices.^49−52^ Studies have demonstrated that optimizing the balance between slipping and sticking can lead to improved morphological properties and enhanced device efficiencies, highlighting the importance of controlling both mechanisms during the film deposition process.^49−52^

Confirming these mechanisms experimentally is challenging due to the atomic-scale complexity involved.

Atomistic modeling, including ab initio and classical molecular dynamics^53−55^ are essential for elucidating growth processes.^56^ Classical methods allow large-scale simulations^57−59^ but require appropriate training/fitting of the model parameters to the material of interest and there are no models available for Si-CsPbBr_3_ systems to date. Ab initio methods are computationally expensive but predictive for all chemical species and provide atomic-level insight into interfacial interactions such as binding energies and charge transfer, enabling an understanding of growth stability and preferred nucleation sites.^56^

In this study, we employ ab initio approach to explore CsPbBr_3_ growth on silicon-based substrates (pure silicon, amorphous silica, and α-quartz). Surprisingly, we find that when the perovskite molecules are put in contact with the 111-silicon surface they breakup into fragments with the formation of Si–Br bonds that sticks on the surface. In contrast, the perovskite unit remains intact on crystalline silicon dioxide, thereby supporting the existence of the “slipping and sticking” mechanisms when the molecules are deposited on amorphous silicon dioxide. By verifying these growth behaviors, we provide key insights into optimizing growth conditions for perovskite-based tandem solar cells, advancing efforts toward highly efficient, silicon-compatible photovoltaic devices.^24−26^

The silicon (111) 7 × 7 surface is a well-characterized reconstruction in semiconductor physics, renowned for its unique atomic arrangement and stability. This structure emerges from the reorganization of silicon atoms at the surface, forming a 7 × 7 unit cell composed of faulted and unfaulted halves, as well as adatom and rest-atom sites. These features collectively enhance the surface’s stability and electronic properties, making it an ideal substrate for the epitaxial growth of metal halide perovskites.^60^ During epitaxial growth, thin crystalline layers form on the silicon substrate with minimal defects. The periodicity and stability of the 7 × 7 reconstruction help reduce lattice mismatches and dislocations during layer growth, supporting the formation of high-quality crystalline films.^61^ This is particularly valuable in photovoltaic applications, where growth on the silicon (111) 7 × 7 surface minimizes interfacial defects and promotes efficient electron transport across heterojunctions.

However, directly modeling the full 7 × 7 unit cell using density functional theory (DFT) is computationally demanding due to the exponential increase in the number of atoms and the resulting number of electrons treated simultaneously, the associated scaling of computational resources, and the complexities involved in accurately capturing electronic interactions. To overcome this challenge, we employed a simplified 2 × 2 four-layer model that effectively captures the essential structural and electronic characteristics of the 7 × 7 surface while significantly reducing computational costs. We plotted Δ*E* versus the number of layers, where Δ*E* = *E*(*i*+1) – *E*(*i*), and both energy values were scaled by the total number of atoms. This approach enabled the calculation of the energy increase when transitioning from layer *i* to layer *i*+1. A cutoff energy of Δ*E* = 10^–3^ Ry was used. Calculations demonstrated that this four-layer atomic model accurately reflects the properties of the 7 × 7 reconstruction, including its lattice parameters and compatibility with materials like CsPbBr_3_.^62^ By using this streamlined model, we can efficiently simulate and analyze the surface interactions and initial steps of growth processes.^63^

We examined the adsorption behavior of three molecular units: CsBr, PbBr_2_, and CsPbBr_3_ commonly introduced as molecular beams in ultrahigh vacuum (UHV) environments. Selected for their relevance to perovskite formation, CsPbBr_3_ is the smallest stoichiometric unit in perovskite film growth. By simulating the adsorption of a first molecular unit (labeled “1” in Table 1) and subsequently a second molecular unit (labeled “2” in Table 1) onto the model Si(111) surface, and by calculating the corresponding binding energies, we gained insights into each molecule’s stability and affinity for the silicon substrate.

**Table 1:** Absorption Energies of the First and Second Units of CsBr, PbBr_2_, and CsPbBr_3_ on Silicon(111)

unit	Δ(CsBr)/eV	Δ(PbBr_2_)/eV	Δ(CsPbBr_3_)/eV
1	1.802	2.547	2.262
2	1.816	2.536	2.606

All molecular units dissociated, forming chemical bonds between the fragments and the substrate, as evidenced by the absorption energies shown in Table 1.

The silicon (111) surface, with its unique atomic and electronic structure, exhibits a strong affinity for bromide ions from the molecular units. Consequently, the CsPbBr_3_ unit dissociates upon adsorption, promoting bromide migration to the silicon surface and leading to the formation of a bromide-modified layer. This surface functionalization alters the local potential, which may adversely affect subsequent adsorption and epitaxial growth processes.

Introducing a second molecular unit—CsBr, PbBr_2_, or CsPbBr_3_—to the silicon surface revealed a consistent binding energy profile (see Table 1), indicating stable adsorption across all species. However, the dissociation of these molecular units presents challenges for epitaxial film formation, as it can disrupt the orderly layering necessary for high-quality perovskite films.

The extensive bromide coverage resulting from this dissociation suggests that while the silicon (111) surface is chemically active, the formation of a bromide-modified layer may hinder the epitaxial growth of perovskite films by altering surface properties unfavorably. A clean Si(111) surface (especially if not fully passivated or hydrogen-terminated) possesses dangling bonds—unpaired electrons on surface Si atoms. These dangling bonds are highly reactive sites. In contrast to bare Si, an SiO_2_ surface is characterized by saturated silicon–oxygen bonds. Most of the oxygen atoms on silica surfaces are already 2-fold-coordinated to silicon, leaving fewer reactive sites or “dangling” bonds. Further investigation is required to determine whether bromide coverage can be controlled or mitigated to support effective film deposition.

To further understand the adsorption dynamics and reaction pathways, we performed Nudged Elastic Band (NEB) calculations on the first adsorbed unit of each molecule (CsPbBr_3_, CsBr, and PbBr_2_) on the silicon surface. The NEB results for CsPbBr_3_, shown in Figure 1, reveal an effectively barrierless path for the initial adsorption step with breaking of molecule and direct formation of new Si–Br or Si–Pb bonds. The same behavior was observed for both CsBr and PbBr_2_ (see Supporting Information Figures S1 and S2), indicating that these molecules similarly follow a direct, momentum-driven (ballistic) adsorption process on the silicon surface. NEB allowed us to map the minimum energy path (MEP), revealing transition states and adsorption energy barriers.

**Figure 1:**
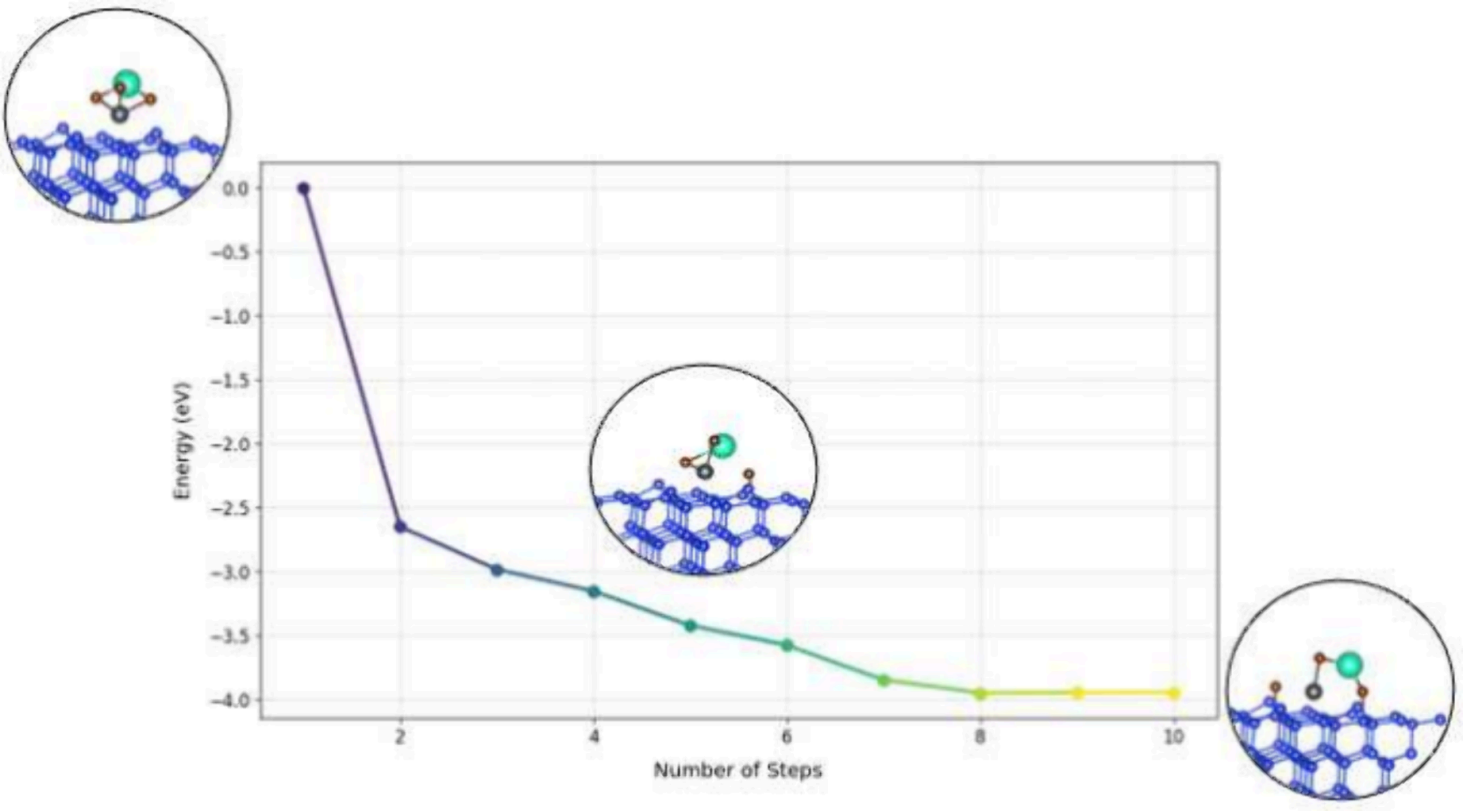
Nudged elastic band (NEB) calculations for CsPbBr_3_ on Si(111). The left inset shows the initial CsPbBr_3_ structure placed far enough from the surface to avoid interaction. The middle inset illustrates the breaking of one Pb–Br bond, with the detached Br atom bonding to the Si surface. The right inset depicts the breaking of a second Pb–Br bond, followed by the attachment of another Br atom to the Si surface.

The results confirm a ballistic reaction path characterized by a continuous decrease in energy as each molecule transitions between stationary points. This direct adsorption process involves the dissociation of the molecule upon surface contact, with bromine atoms bonding to and functionalizing the silicon surface. The ballistic pathway is effectively barrier-free and driven by the initial momentum of the molecule.

Unlike the “slip and stick” mechanism typically associated with epitaxial growth, the initial adsorption process of CsPbBr_3_ on silicon does not follow this pattern when using pure silicon surfaces. Pure silicon lacks the necessary surface functional groups to facilitate orderly layer formation, leading to irregular or nonuniform deposition. Epitaxial growth only commences once the silicon surface is fully functionalized with bromine atoms. This bromine functionalization creates specific binding sites that promote the orderly adsorption of CsPbBr_3_ molecules, potentially starting effectively with the adsorption of the second molecule onward. While this functionalization step is crucial for enabling CsPbBr_3_ deposition, it may simultaneously restrict the formation of highly ordered layers, which are essential for achieving optimal performance in tandem solar cells. Additionally, ab initio computational methods, which rely on first-principles calculations of atomic interactions, often struggle to capture large-scale layer formation due to inherent system-size and time scale constraints. However, these limitations do not significantly affect this study because it focuses on local atomic-level mechanisms of adsorption, which can be accurately probed using smaller, representative surface models. By examining these essential interactions, the work still provides a robust understanding of the functionalized epitaxial growth on bromine-functionalized silicon surfaces.

Inspired by very recent experimental results^45^ we examined interactions between molecular species and amorphous silica.

To mimic realistic conditions we considered surfaces passivated by hydroxyl groups with varying densities (i.e., 15, 24, 45, 54, and 74 OH per unit area) to capture realistic surface variations.

In amorphous silica, adsorption differs notably from silicon (111): neither the first nor the second molecular units dissociated upon adsorption, indicating a weaker interaction; see absorption energies in Table 2. Molecular species retained an average distance of approximately 3.5 Å from surface oxygen atoms, suggesting the absence of a strong covalent bonding.

**Table 2:** Absorption Energies of First Unit (1) and Second Unit (2) of CsBr, PbBr_2_, and CsPbBr_3_ on Different Surfaces of Amorphous Silica: 15OH, 24OH, 45OH, 54OH, and 74OH

	Δ/eV					
	CsBr(1)	PbBr_2_(1)	CsPbBr_3_(1)	CsBr(2)	PbBr_2_(2)	CsPbBr_3_(2)
15OH	0.567	0.477	0.152	1.476	0.784	0.978
24OH	0.841	0.508	0.158	0.895	0.432	1.160
45OH	0.614	0.264	0.353	1.164	0.766	0.756
54OH	0.351	0.478	0.495	0.809	0.716	0.749
74OH	0.639	0.349	0.311	1.259	0.521	0.502

Here, we also performed NEB calculations on the first unit of CsPbBr_3_ adsorbed on different amorphous silica surfaces. Our results revealed a diffusive pathway, whose energy profiles are shown in Figure 2 with corresponding atomistic structures depicted in Figure 3. The variations in the energy barriers reflects the disorder of the amorphous layer and depends on the local atomistic morphology and surface roughness.

**Figure 2:**
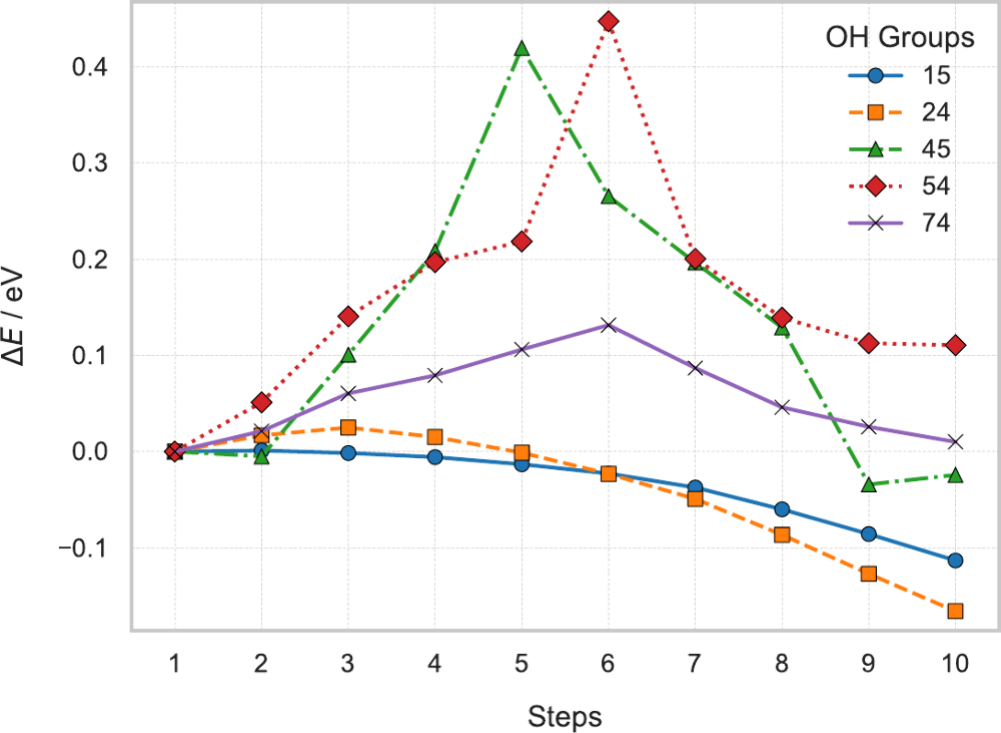
Nudged elastic band (NEB) calculations for CsPbBr_3_ on amorphous silica.

**Figure 3:**
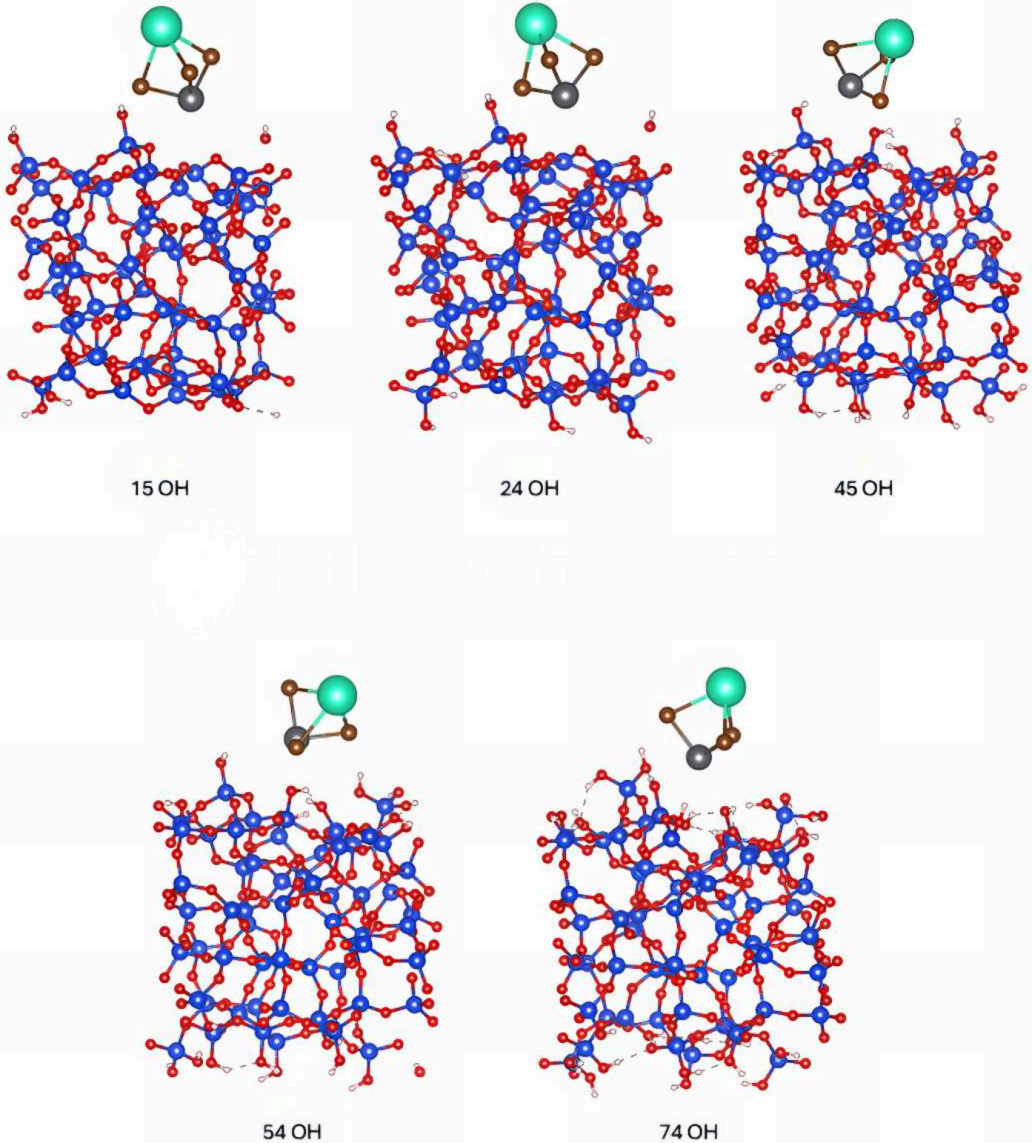
Amorphous silica substrates with CsPbBr_3_.

The calculations indicate that the CsPbBr_3_ unit is capable of diffusing on the amorphous silica surface while maintaining its structural integrity. The calculated energy barriers are small (values are in the range 0–0.4 eV) indicating high diffusivity at room temperature. By assuming a prefactor ν of 10^12^ Hz and displacement length λ of 6 Å, we can estimate diffusivity *D* ≈ *νλ*^2^*e*^–*E*/*kT*^ ranging within 10^–9^–10^–5^ cm^2^ s^–1^. These findings are consistent with the “slip and stick” mechanism previously proposed by De Padova et al.^45^

To determine whether the absorption results are influenced by the amorphous morphology or the chemical nature of the silicon dioxide substrate, we also examined absorption on the crystalline [001] surface of α-quartz. Table 3 presents the absorption energies for the first and second units of CsBr, PbBr_2_, and CsPbBr_3_ on this crystalline surface. The absorption energies are comparable to those observed for the amorphous substrate, indicating similar binding mechanisms and energy profiles. Additionally, the bond distances between the substrate and the molecular species are close to those in the amorphous case, as shown in Table 3.

**Table 3:** Absorption Energies of the First and Second Units of CsBr, PbBr_2_, and CsPbBr_3_ on the [001] Surface of α-Quartz

unit	Δ(CsBr)/eV	Δ(PbBr_2_)/eV	Δ(CsPbBr_3_)/eV
1	0.259	0.294	0.069
2	0.693	0.304	0.331

Furthermore, NEB calculations for CsPbBr_3_ on the [001] α-quartz surface identify a minimum-energy path between adjacent adsorption sites with an exceptionally low barrier of about 0.02 eV (Figure 4). This small barrier without the breaking of molecule and formation of new Si–Br or Si–Pb bonds indicates that CsPbBr_3_ diffuses easily under typical conditions, supporting the conclusion that its surface motion is predominantly governed by a diffusive mechanism. This observation supports the ”slip and stick” mechanism,^45^ highlighting the significant dependence of the energy barrier on surface roughness. The contrast between silica and silicon substrates also indicates variability in adsorption behavior based on surface composition and morphology. Specifically, amorphous silica exhibits more passive interactions compared to the reactive silicon (111) surface. These findings suggest that molecular beam adsorption mechanisms are substrate-dependent, a critical factor for understanding and optimizing growth processes in semiconductor applications.

**Figure 4:**
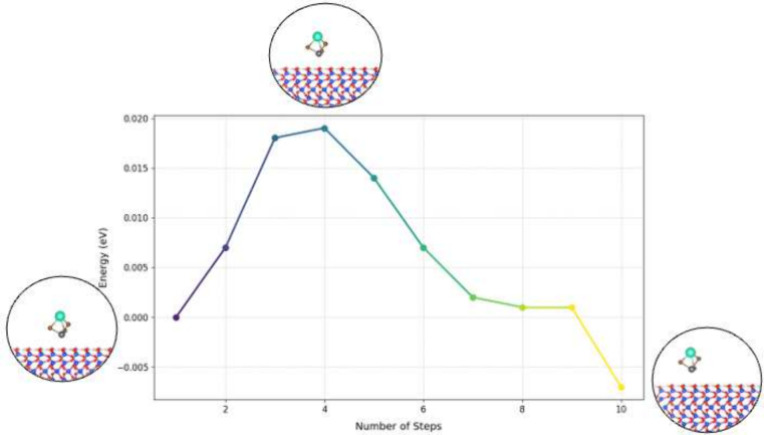
Nudged elastic band (NEB) calculations for CsPbBr_3_ on the [001] surface of α-quartz. The left inset shows the initial local minimum on the surface where CsPbBr_3_ structure is placed. The middle inset illustrates that no bonds in the molecular structure are broken as it moves across the surface at the peak of the energy barrier. The right inset shows the same structure displaced to a different local minimum (i.e., site) relative to its initial position.

The results presented here can be summarized by scheme showed in Figure 5. They support the occurrence of CsPbBr_3_ epitaxial growth on amorphous silica via a “slip and stick” mechanism. In this process, precursors bind to the surface without breaking up and easily diffuse on the surface, which allows for crystal growth. Unlike the highly reactive silicon (111) surface, which induces molecular dissociation upon adsorption, amorphous silica preserves the integrity of adsorbed species, preventing bond-breaking events. The chemically inert nature of amorphous silica supports surface saturation through low-barrier diffusion and enables the formation of uniform thin films, which is essential for seamlessly integrating multiple photoactive layers in tandem solar cells. Its reduced reactivity minimizes unwanted chemical interactions, providing a stable, noninterfering interfacial layer that enhances both durability and efficiency in tandem devices. In contrast, the pronounced reactivity of the silicon (111) surface can disrupt the structural and electronic continuity of stacked layers, challenging device stability.

**Figure 5:**
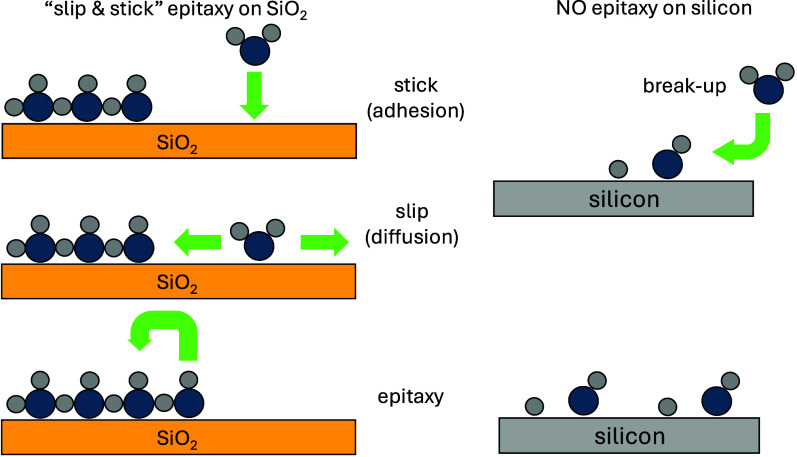
Scheme of epitaxial growth on SiO_2_ and silicon.

Thus, the “slip and stick” mechanism on amorphous silica underscores its suitability as an ideal passivation layer, supporting the precise, layer-by-layer assembly needed to achieve high-efficiency tandem solar cells.

## Supporting Information Available

Computational details and pictures of NEB for CsBr and PbBr_2_ on Si(111) (PDF)
