# Correction to “Amorphous Silica Interlayer Unlocks Direct Epitaxial Growth of CsPbBr_3_ on Silicon via Slip-and-Stick Mechanism”

**DOI:** 10.1021/acs.jpclett.5c00586

**Published:** 2025-05-19

**Authors:** Christian Tantardini, Simone Argiolas, Paola De Padova, Boris I. Yakobson, Aldo Di Carlo, Alessandro Mattoni

The original version of this Article contained several errors in the cited literature. References 14–21, 23, 27–29, 31–34, 43, 46,^49–52^ , and^60–63^ have been replaced. Following is the corrected reference list.^1–63^
