# Supplementary material for: Amorphous Silica Interlayer Unlocks Direct Epitaxial Growth of CsPbBr3 on Silicon via Slip-and-Stick Mechanism
Source: J Phys Chem Lett. 2025 Feb 26;16(9):2385–92. doi: 10.1021/acs.jpclett.4c03705 (PMC11891965; doi:10.1021/acs.jpclett.4c03705)
Supplement: Supplementary file 1 — jz4c03705_si_001.pdf [file jz4c03705_si_001.pdf]

Supplementary Information
Amorphous Silica Interlayer Unlocks Direct Epitaxial Growth of $CsPbBr_3$ on Silicon via Slip-and-Stick Mechanism

Christian Tantardini,[*,†,‡] Simone Argiolas,[†,¶] Paola De Padova,[§] Boris Yakobson,[§,‡] Aldo Di Carlo,[§] and Alessandro Mattoni[*,†]

†*CNR - Istituto Officina dei Materiali (IOM) Cagliari, Cittadella Universitaria, Monserrato (CA), 09042, Italy.*

‡*Department of Materials Science and NanoEngineering, Rice University, Houston, Texas 77005, United States of America.*

¶*Dipartimento di Fisica, Università degli Studi di Cagliari, Cittadella Universitaria, Monserrato (CA), 09042, Italy.*

§*National Research Council, Institute of Structure of Matter (CNR - ISM), Via Fosso del Cavaliere, 100, 00133 Roma, Italy.*

E-mail: christiantantardini@ymail.com; mattoni@iom.cnr.it

Computational Details

To study the growth of perovskite materials on silicon-based surfaces, we employed *ab-initio* methods to capture detailed material properties and interfacial interactions. A specific validation approach was introduced to assess the reliability of results obtained with the plane-

wave basis. Initially, we optimized the structures using an all-electron approach, allowing all atoms to relax, including both the molecular fragment of $CsPbBr_3$ and the surfaces of Si[111] and (001) surface of α-quartz. This approach enabled precise optimization of atomic positions across the full system. We then compared these all-electron geometries to those optimized using plane-waves approach, where the substrate was fixed during optimization. These comparisons showed only minor atomic displacements on the substrate, in the order of 10^{-4} Å, confirming that fixing the substrate in plane-wave calculations does not compromise accuracy and is appropriate for allowing only the molecular fragment to move during the optimization process.

For electronic structure calculations, we utilized FHI-aims[1–5]for all electron calculations where the total energy convergence threshold was set to 10^{-4} eV, and the interatomic force convergence threshold was set to 10^{-3} eV/Å; while for plane-waves calculations we used Quantum Espresso version 7.3[6,7] with Density Functional Theory (DFT) using the Perdew-Burke-Ernzerhof (PBE) exchange-correlation functional.[8] Projector augmented-wave (PAW) pseudopotentials from PseudoDojo[9] described ion-electron interactions, with a kinetic energy cutoff of 20 Ry. Self-consistent field (SCF) convergence was achieved when the total energy changed by less than 10^{-6} Ry and the interatomic forces by less than 10^{-5} Ry/atom.

To model the (111) silicon slab, a convergence study indicated that a four-layer slab was sufficient. A Python script generated slabs from the conventional silicon cell with Hermann-Mauguin space group Fd3m, using a Monkhorst-Pack k-point mesh density of $4 \times 4 \times 1$ and a vacuum layer of 15 Å along the c-axis. We then simulated the adsorption of molecular species, such as CsBr, $PbBr_2$, and $CsPbBr_3$, on a $2 \times 2 \times 1$ supercell of the four-layer Si(111) slab using a Monkhorst-Pack grid density of $2 \times 2 \times 1$, keeping silicon atomic positions fixed.

For the crystalline silica surface, we modeled the most stable (001) surface of α-quartz (space group P3121).[10–13] Under ambient conditions, this surface converts to silanol groups upon reaction with atmospheric water at under-coordinated sites. The structure used was a 4×4 supercell of (001) α-quartz with 300 atoms, representing geminal silanols. This

fixed structure was based on all-electron calculations from Iliaš and Pershina.[14] Adsorption calculations for CsBr, $PbBr_2$, and $CsPbBr_3$ on this surface used a Monkhorst-Pack k-point grid density of $2 \times 2 \times 1$.

To represent amorphous silica, we employed the Ugliengo model,[15] derived from Cristobalite and consisting of 192 atoms. The Ugliengo model was generated by high-temperature molecular dynamics, followed by cooling, saturation, and *ab-initio* relaxation using the CRYSTAL code[16] with the B3LYP functional[17] and the 6-31g(d,p) basis set.[18,19] Variants with 15, 24, 45, 54, and 74 OH groups were generated. We calculated adsorption on each amorphous silica model, keeping positions fixed and applying a Monkhorst-Pack grid density of $2 \times 2 \times 1$.

We have uploaded in ZENODO at the link `wwww.XyZ` all the input and output got from FHI-aims and Quantum Espresso. Here, below we have presented the fundamental data to complement what we have proposed in the main text.

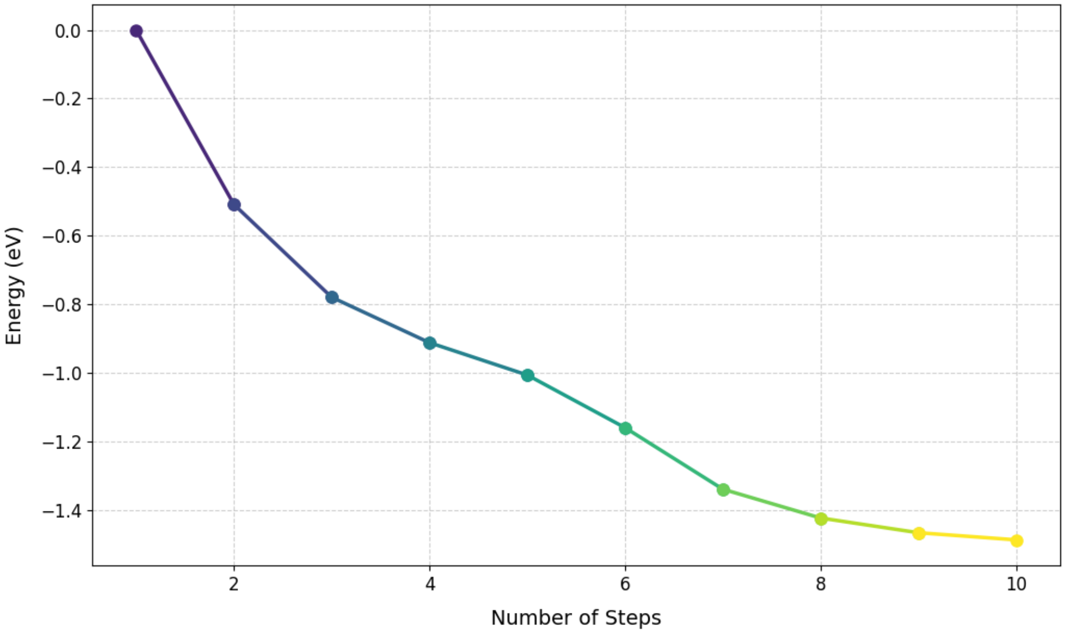

Figure S1: Nudged Elastic Band (NEB) calculations of one unit of CsBr on Si(111) surface.

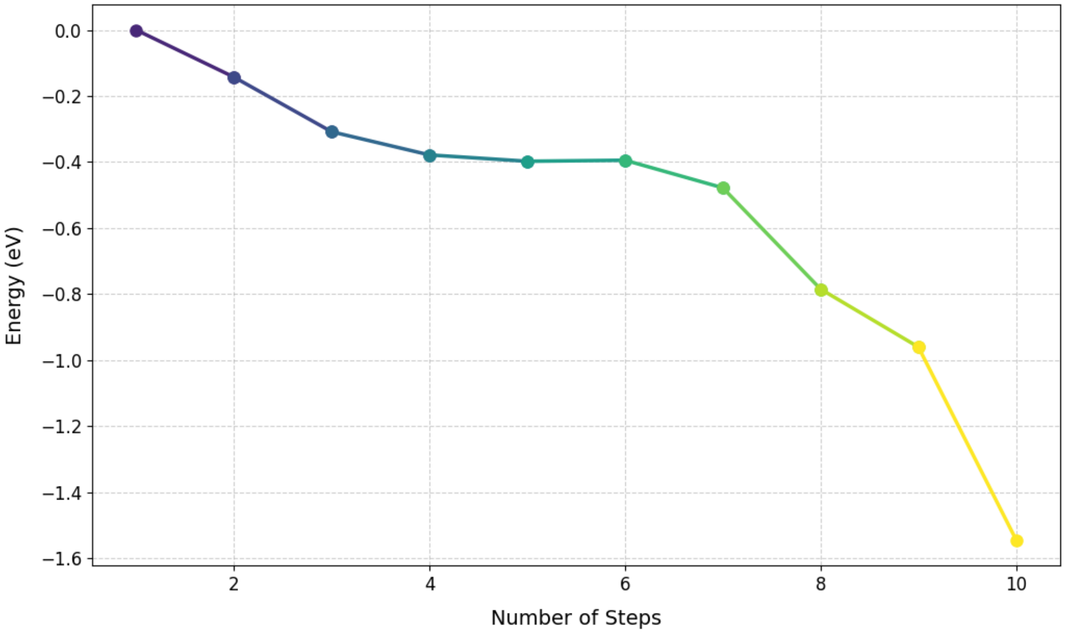

Figure S2: Nudged Elastic Band (NEB) calculations of one unit of $PbBr_2$ on Si(111) surface.

References

(1) Blum, V.; Gehrke, R.; Hanke, F.; Havu, P.; Havu, V.; Ren, X.; Reuter, K.; Scheffler, M. Ab initio molecular simulations with numeric atom-centered orbitals. *Computer Physics Communications* **2009**, *180*, 2175–2196.

(2) Havu, V.; Blum, V.; Havu, P.; Scheffler, M. Efficient O(N) integration for all-electron electronic structure calculation using numeric basis functions. *Journal of Computational Physics* **2009**, *228*, 8367–8379.

(3) Knuth, F.; Carbogno, C.; Atalla, V.; Blum, V.; Scheffler, M. All-electron formalism for total energy strain derivatives and stress tensor components for numeric atom-centered orbitals. *Computer Physics Communications* **2015**, *190*, 33–50.

(4) Levchenko, S. V.; Ren, X.; Wieferink, J.; Johanni, R.; Rinke, P.; Blum, V.; Scheffler, M. Hybrid functionals for large periodic systems in an all-electron, numeric atom-centered basis framework. *Computer Physics Communications* **2015**, *192*, 60–69.

(5) Ihrig, A. C.; Wieferink, J.; Zhang, I. Y.; Ropo, M.; Ren, X.; Rinke, P.; Scheffler, M.; Blum, V. Accurate localized resolution of identity approach for linear-scaling hybrid density functionals and for many-body perturbation theory. *New Journal of Physics* **2015**, *17*, 093020.

(6) Giannozzi, P.; Baroni, S.; Bonini, N.; Calandra, M.; Car, R.; Cavazzoni, C.; Ceresoli, D.; Chiarotti, G. L.; Cococcioni, M.; Dabo, I.; others QUANTUM ESPRESSO: a modular and open-source software project for quantum simulations of materials. *Journal of physics: Condensed matter* **2009**, *21*, 395502.

(7) Giannozzi, P.; Andreussi, O.; Brumme, T.; Bunau, O.; Nardelli, M. B.; Calandra, M.; Car, R.; Cavazzoni, C.; Ceresoli, D.; Cococcioni, M.; others Advanced capabilities for materials modelling with Quantum ESPRESSO. *Journal of physics: Condensed matter* **2017**, *29*, 465901.

(8) Perdew, J. P.; Burke, K.; Ernzerhof, M. *Phys. Rev. Lett* **1996**, *77*, 3865–3868.

(9) Jollet, F.; Torrent, M.; Holzwarth, N. Generation of Projector Augmented-Wave atomic data: A 71 element validated table in the XML format. *Computer Physics Communications* **2014**, *185*, 1246–1254.

(10) Rimola, A.; Costa, D.; Sodupe, M.; Lambert, J.-F.; Ugliengo, P. Silica surface features and their role in the adsorption of biomolecules: computational modeling and experiments. *Chemical reviews* **2013**, *113*, 4216–4313.

(11) Krüger, P.; Pollmann, J. Ab initio calculations of Si, As, S, Se, and Cl adsorption on Si(001) surfaces. *Phys. Rev. B* **1993**, *47*, 1898–1910.

(12) Somasundaran, S. *Encyclopedia of Surface and Colloid Science, 2nd ed.*; CRC Press, Boca Raton, 2006.

(13) Papirer, E. *Adsorption on Silica Surfaces, Surfactant Science Series*; Taylor & Francis, Boca Raton, New York, 2000.

(14) Iliaš, M.; Pershina, V. Theoretical predictions of properties and adsorption behaviour of a superheavy element Ts and its lighter homolog At, and of their various gas-phase compounds, on hydroxylated quartz surfaces from periodic DFT calculations. *Molecular Physics* **2024**, *0*, e2363408.

(15) Ugliengo, P.; Viterbo, D.; Chiari, G. MOLDRAW: Molecular graphics on a personal computer. *Zeitschrift für Kristallographie - Crystalline Materials* **1993**, *207*, 9–24.

(16) Erba, A.; Desmarais, J. K.; Casassa, S.; Civalleri, B.; Donà, L.; Bush, I. J.; Searle, B.; Maschio, L.; Edith-Daga, L.; Cossard, A.; Ribaldone, C.; Ascrizzi, E.; Marana, N. L.; Flament, J.-P.; Kirtman, B. CRYSTAL23: A Program for Computational Solid State Physics and Chemistry. *Journal of Chemical Theory and Computation* **2023**, *19*, 6891–6932.

(17) Becke, A. D. Density-functional thermochemistry. III. The role of exact exchange. *The Journal of Chemical Physics* **1993**, *98*, 5648–5652.

(18) Del Bene, J. E.; Person, W. B.; Szczepaniak, K. Properties of Hydrogen-Bonded Complexes Obtained from the B3LYP Functional with 6-31G(d,p) and 6-31+G(d,p) Basis Sets: Comparison with MP2/6-31+G(d,p) Results and Experimental Data. *The Journal of Physical Chemistry* **1995**, *99*, 10705–10707.

(19) Hehre, W. J.; Ditchfield, R.; Pople, J. A. Self—Consistent Molecular Orbital Methods. XII. Further Extensions of Gaussian—Type Basis Sets for Use in Molecular Orbital Studies of Organic Molecules. *The Journal of Chemical Physics* **1972**, *56*, 2257–2261.
